# Factor XIII Subunit A in the Skin: Applications in Diagnosis and Treatment

**DOI:** 10.1155/2017/3571861

**Published:** 2017-08-15

**Authors:** Lilla Paragh, Daniel Törőcsik

**Affiliations:** Department of Dermatology, Faculty of Medicine, University of Debrecen, Debrecen, Hungary

## Abstract

The role of factor XIII subunit A (FXIII-A) is not restricted to hemostasis. FXIII-A is also present intracellularly in several human cells and serves as a diagnostic marker in a wide range of dermatological diseases from inflammatory conditions to malignancies. In this review, we provide a guide on the still controversial interpretation of dermal cell types expressing FXIII-A and assess the previously described mechanisms behind their accumulation under physiological and pathological conditions of the human skin. We summarize the intracellular functions of FXIII-A as well as its possible sources in the extracellular space of the dermis with a focus on its relevance to skin homeostasis and disease pathogenesis. Finally, the potential role of FXIII-A in wound healing, as a field with long-term therapeutic implications, is also discussed.

## 1. Introduction

Factor XIII (FXIII), fibrin stabilizing factor, is an enzyme consisting of two subunits: an A (FXIII-A) and a B subunit (FXIII-B) that can form a tetrameric complex in the plasma (pFXIII-A) with two of each subunit (FXIII-A_2_B_2_). As a member of the transglutaminase family, FXIII crosslinks fibrin residues to produce the mature clot in blood coagulation [1]. In addition to being a pivotal member of the coagulation cascade, FXIII-A is also found intracellularly (cFXIII-A) in various cells all around the body. Besides megakaryocytes and platelets, monocytes [2], macrophages [3], dendritic cells (DCs) [4], fibroblasts [5], mast cells [6], and sebocytes [7], all with an important role in the homeostasis of the skin, have been listed as FXIII-A-producing cells. Thus, FXIII-A is of interest in not only the fields of hemostasis but also cellular expression and skin biology. Therefore in this review, besides giving a general overview on FXIII-A production and its presence in the intracellular and extracellular space, we also aim to summarize our current knowledge on the role of FXIII-A in the (patho)physiology of the skin and discuss its possible cutaneous therapeutic applications. (List of abbreviations of certain forms of factor XIII used in the article is shown in Abbreviations.)

## 2. FXIII-A in the Circulation

FXIII, as mentioned before, is a member of the transglutaminase family. In the plasma, FXIII is present as a tetramer where FXIII-A is the catalytic enzyme and FXIII-B is a glycoprotein responsible for inhibiting FXIII-A upon binding to it. Only approximately 1% of FXIII-A exists in a free form in the plasma. FXIII-A upon activation (FXIII-A_2_^*∗*^) crosslinks the *γ*-glutamyl and *ε*-lysyl residues of connected polymerized fibrin chains to produce the mature clot during hemostasis [1]. For more details regarding their interaction and role in blood coagulation, we refer to the excellent reviews [8–10].

While FXIII-B is exclusively produced by the liver [11] and is only present in the circulation, FXIII-A can also be found in the extracellular space and in the cytoplasm of various cells. Therefore, we are focusing our review on the role of FXIII-A. pFXIII-A has long been thought to originate in bone marrow, mainly produced by megakaryocytes and platelets. However, the findings of Poon et al. suggested that other cells might also contribute to pFXIII-A levels. During bone marrow ablation, they noted only a 25% reduction in pFXIII-A levels in contrast to a 90% reduction in platelet count. The reduction of pFXIII-A levels was also less than expected in thrombocytopenic patients, further supporting the presence of a nonthrombopoietic source of pFXIII-A [1, 2, 12]. Studies by Griffin et al., using a model where mice floxed in coding exon 7 of the FXIII-A gene* (F13A1)* were crossed with mice transgenic for* Pf4-Cre-*recombinase (thrombopoietic deletion) or* Cd11b-Cre-*recombinase (myeloid deletion), raised the possibility that a unique Pf4-dependent progenitor cell is the major source of the plasma pool, which was independent of thrombopoietin receptor. While, in* F13A1*^*fl/fl*^*-Pf4-cre* mice, FXIII-A plasma activity was decreased by 85% and absent in platelets, in* F13A1*^*fl/fl*^*-CD11b-cre* mice plasma activity decreased by 40%. Interestingly,* F13A1 *mRNA levels also decreased in the aorta (91.6%) and heart (99.2%) of* F13A1*^*fl/fl*^*-Pf4-cre* mice but showed no reduction in the heart and a 54.6% reduction in the aorta tissue samples of* F13A1*^*fl/fl*^*-CD11b-cre* mice [13].

## 3. Extracellular FXIII-A

Because FXIII-A lacks a signal sequence for secretion, for a long time, the only possible explanation was that ecFXIII-A either was from the circulation or originated from dying cells. This was supported by in vitro findings that FXIII-A could not be detected in the culture medium when it was expressed in baby hamster kidney cells [10]. However, Cordell et al. suggested nonclassical secretion by showing that FXIII-A in macrophages was associated with podosomes and was present in intracellular vesicles associated with Golgi matrix protein-130 (GM-130), which is involved in the delivery of proteins to the plasma membrane [14]. Interestingly, under specific conditions, FXIII-A can also appear on the cell surface where it could exert its transglutaminase activity and play a role in modulating cell adhesion [15].

Based on these findings, FXIII-A-positive macrophages were implicated in the elevation of ecFXIII levels both in the bronchoalveolar lavage fluid of patients with asthma [16] and in tissue samples of patients with chronic rhinosinusitis with nasal polyps [17]. Considering the known substrate profile of FXIII-A, such as fibronectin, vitronectin, osteopontin, thrombospondin, and certain adhesive glycoprotein components of the extracellular matrix (ECM), which will be discussed later in this review, the alterations in the ecFXIII-A levels might contribute to tissue repair in these diseases. Although the correlation between the levels of ecFXIII-A and the increased number of FXIII-A-positive macrophages corroborated that cFXIII-A could be a potent contributor to ecFXIII-A levels [15], questions as to which of the FXIII-A producing cell types could indeed contribute to ecFXIII-A and the stimuli behind its possible secretion are yet to be answered. Based on these findings, future studies are also needed in the field of dermatology to examine the correlation of ecFXIII-A with FXIII-A-positive cells within various skin lesions. While using mouse models with selective ablation of the FXIII-A-positive skin-resident macrophages, their contribution to ecFXIII-A (and perhaps to pFXIII-A) levels could be assessed as well.

## 4. Intracellular FXIII-A

Whereas, in early embryonic life, the cells positive for cFXIII-A are the mesenchymal histiocytes and hepatocytes [18], in adult life, in addition to monocytes and tissue macrophages [3], cFXIII-A was detected also in DCs [4], fibroblasts [5], sebocytes [7], and mast cells of the skin and recently in the subcutaneous preadipocytes [19]. Although it is outside the scope of this review, osteoblasts, chondrocytes [20, 21], and cornea cells [22] should also be listed here as FXIII-A-producing cells [23, 24]. Confirming that the intracellular Ca^2+^ concentration is sufficient for its activation within the cell as shown in platelets and monocytes [8], cFXIII-A was also suggested to play a role in various intracellular (intracytoplasmic and intranuclear) processes [25].

### 4.1. Macrophages

Being the first cell type in which cFXIII-A was detected more than 30 years ago [26], the circulating blood monocytes/macrophages provide an excellent model for the examination of the possible roles of cFXIII-A. It was shown that cFXIII-A is present from a very early stage of monocyte differentiation (monoblasts in the bone marrow) [27, 28] to the macrophages of connective tissue and serous cavities [29]. Based on these findings, FXIII-A became interpreted as a marker protein of the cell line. Actin and myosin [30], the two major elements of the cell cytoskeletal locomotory system, were the first identified intracellular substrates, followed by vinculin [31], the small heat shock protein HSP27 [32], and thymosin beta-4 [33]. All these molecules have a significant role in cytoskeletal remodeling [8]. cFXIII was also suggested to participate in the phagocytosis of certain particles. Fcg and complement receptor-mediated uptake of sensitized erythrocytes and complement-coated yeast particles were greatly diminished in monocytes of FXIII-deficient patients. The phagocytic functions of cultured monocytes/macrophages showed changes alongside FXIII-A mRNA expression and protein synthesis [34]. In addition, cFXIII-A can also induce the activation and mobilization of monocytes from the splenic reservoir in response to angiotensin II binding its receptor (AT1), which can activate cFXIII-A that in return dimerizes AT1 [35].

Interestingly, FXIII-A was also shown to translocate to the nucleus of differentiating macrophages where it may also exert its enzymatic activity. It still needs to be elucidated if this is behind the altered gene expression profile of FXIII-A-deficient macrophages or a cytoplasmic activity targeting unidentified proteins. The significant changes in genes involved in wounding, immune processes, and ECM formation show that cFXIII-A is involved in a wide range of cellular processes, including participating in gene expression regulation of the key functions of macrophages [15]. However, we also showed that the presence of cFXIII-A was not ubiquitous in macrophages but marked a specific activated status, the so-called alternative activation. Such activated macrophages are characteristic to the healthy skin and dominant in pathological conditions where inflammation is not related to infection. These are conditions, such as wounding or tumor development, that display ECM formation and where cFXIII-A expression could have a significant diagnostic value that we will introduce in more detail later in the dermatopathology section. The key cytokine driving macrophages toward the alternative polarization is IL-4. IL-4 was shown to be the strongest inducer of FXIII-A gene expression and protein levels within macrophages so far [15]. On the contrary, in infectious lesions, such as tuberculosis, TNF*α* and IFN*γ* are the cytokines behind the classical activation of macrophages and in parallel inhibit the expression of cFXIII-A [34]. This interesting finding could explain, at least partially, why FXIII-A-deficient patients have no reported increased susceptibility to infectious diseases.

### 4.2. Dendritic Cells

In addition to macrophages, FXIII-A was identified in a great variety of cell types. However, the question of antibody specificity was put forward in many cases. The validity of the term “FXIII-A dendrocytes,” which has been widely accepted and is still used [36], is one of the prime examples of such debates. A detailed characterization of dermal FXIII-A-positive cells by Zaba et al. highlighted that FXIII-A could be detected only in macrophages. Cells capable of antigen presentation in the skin, which is the hallmark of dendritic cells (DCs), lacked FXIII-A [37]. A possible explanation for the discrepancies between ex vivo and in vitro findings could be that IL-4 in combination with GM-CSF is a widely used but also challenged [38] cytokine combination for in vitro DC differentiation. In such models, FXIII-A-deficient DCs showed a reduced chemotactic response to CCL19 and impaired cell motility [39].

Based on these data, further studies are needed to determine if the presence of cFXIII-A is just a “side-product” in in vitro differentiated DCs, due to its previously described induction by IL-4, or if there are certain DC subsets expressing FXIII-A also under in vivo conditions.

### 4.3. Mast Cells

Recently, interest in mast cells as FXIII-A-producing cells [6] that could contribute to ecFXIII-A and perhaps to pFXIII levels has grown. Using immunogold labeling and electron microscopy, the double staining for FXIII-A and tryptase showed that, in sections of drug-induced acute urticaria, the two proteins colocalized within the granules of mast cells. Interestingly, the granules were also detected in dermal DCs and in endothelial cells of postcapillary venules, suggesting a mast cell, dermal dendrocyte/endothelial cell interaction in urticaria [40, 41]. Additionally, after IgE mediated activation of bone marrow-derived cultured mast cells (BMCMCs), cFXIII-A was found to be one of the most abundant proteins in the proteome. However, in mast cell-deficient mice, pFXIII and activity levels were increased in correlation with reduced bleeding times. The root of these changes was that the human chymase and mouse mast cell protease-4 (the mouse homologue of human chymase) could downregulate FXIII-A via proteolytic degradation. Thus, deficiency of the chymase led to increased FXIII-A amounts and activity and reduced bleeding times in homeostatic conditions and during sepsis. Mast cells are ever-evolving cell types not just in urticaria but also in other dermatological conditions such as psoriasis. It is of crucial importance to further confirm the role of mast cell-derived FXIII-A in human disease settings [6].

### 4.4. Fibroblasts, Sebocytes, and Keratinocytes

FXIII-A positivity of fibroblasts [5], keratinocytes, and sebocytes turned out to be dependent on the applied antibody. A recent study found that the clone AC-1A1 mouse monoclonal antibody for FXIII-A was a useful tool for staining sebocytes with increased proliferative activity. It suggested that, in the future, this antibody can be used in the diagnosis of sebocyte-related malignancies [7]. Using the same antibody, keratinocytes were also positive showing a correlation with their maturation. However, using different antibodies for FXIII-A, we did not detect FXIII-A-positive sebocytes or keratinocytes in the examined specimens, which is in line with other authors.

### 4.5. (Pre)adipocytes

FXIII-A was also shown to negatively regulate adipogenesis in mouse white adipose tissue and in differentiating 3T3-L1 preadipocytes. FXIII-A on the surface of the studied preadipocytes contributed to cell-matrix interactions by promoting the assembly of fibronectin from plasma into the preadipocyte extracellular matrix. Modulation of cytoskeletal dynamics induced the proliferation of the cells and at the same time inhibited their differentiation into lipid-accumulating mature adipocytes. FXIII-A, via the same pathway, might also be crucial in the transformation of embryonic fibroblasts into adipocytes [19]. More and more evidence supports that subcutaneous adipose tissue is in direct communication with the dermis through altering local inflammation and defense mechanisms against pathogens [42]. It is of interest to address subcutaneous adipose tissue and its FXIII-A levels in various inflammatory skin lesions.

## 5. Application of FXIII-A in Dermatopathology

Shortly after the discovery that cFXIII-A is expressed in macrophages of various origin throughout the body [26, 29, 43], its detection has been implicated in dermatological diagnosis and a heterogenic population of FXIII-A-positive dermal dendritic cells was also described [36, 44].

Cerio et al. detected “FXIII-A dendrocytes” both in the neonatal foreskin and normal adult skin mainly in the upper dermis and in the papillary dermis surrounding vessels [45]. Due to the limits of the available functional testing of such cells both in vivo and ex vivo at that time, the term “FXIII-A dendrocyte” was derived from the spindle shape appearance of such cells rather than from their potential to present antigens. As we have previously detailed, with the improvement of available tools and in our knowledge of the differences between macrophages and DCs, it is more likely that FXIII-A positivity indicates alternative activation of macrophages [34, 37].

In the next section, we aim to systematically describe diseases with FXIII-A-positive cells, using the terms for the identified cell types according to original publications.

### 5.1. FXIII-A in Granulomatous Diseases

Granulomas are characteristic to diseases in which the immune system is incapable of eliminating the antigen that can be of pathogen or of noninfectious origin such as lipids or foreign bodies. Although T cells and DCs are also involved in granuloma formation, the key cells leading to the well-recognized histological changes are the macrophages.

Our findings that FXIII-A marks the alternative activation pathway in macrophages is best reflected in diseases of granuloma annulare (GA) and necrobiosis lipoidica (NL), which are the prime examples of noninfectious, nonmalignant inflammatory skin diseases with granuloma formation. Although the activating stimuli behind the symptoms are not known, (connective tissue disorder, impaired local circulation, and lack of anti-inflammatory signaling affecting the macrophages), there is a remarkable change in ECM remodeling. Our results demonstrated that the macrophages in both diseases, expressed both FXIII-A and CD206 (also a widely accepted marker for detecting alternatively activated macrophages). Moreover, we also found that FXIII-A-positive cells formed distinct populations from both CD11c and CD1a expressing cells in GA and in NL [25], in which markers were convincingly shown to detect DCs in the healthy skin [37]. Altogether, besides demonstrating that FXIII-A is a useful tool to mark alternatively activated macrophages also in pathological conditions such as GA and NL, we also proposed to integrate the classification by Zaba et al. for macrophages and DCs into dermatopathology [25]. Importantly, further characterization of FXIII-A-positive cells revealed that the cells expressing FXIII-A also expressed the macrophage marker CD163, whereas only 60% coexpressed CD68, a marker that has been also widely used to detect macrophages. To explain these findings, it should be kept in mind that the skin is a dynamic system and therefore it would be an oversimplification to search for such polarized conditions as described in in vitro conditions in any given disease. Therefore these findings further highlight the importance of multiple labeling in cases where the characterization of the macrophage populations is essential in setting up a diagnosis. To allow further conclusions and new perspectives in its dermatopathological application, we emphasize the importance of using multiple immunohistochemical markers in the description of FXIII-A-positive cells. We have collected the markers that have been used so far in combination with FXIII-A in Table 1.

As we introduced previously, on the other extreme of macrophage polarization is classical activation, which marks the presence of TNF*α* and/or IFN*γ* in the inflammatory environment. These cytokines are typical in pathogen-associated lesions such as tuberculosis granulomas caused by* Mycobacteria* but are also the key cytokines in sarcoidosis, a disease lacking any pathogen. Supporting our previously detailed in vitro findings, macrophages were FXIII-A negative in the inflammatory foci of both tuberculosis and leprosy, just as in sarcoidosis [46]. Interestingly, FXIII-A-positive macrophages could still be found near the granulomas. This finding can be explained with the possible commitment of FXIII-A-positive resident macrophages to keep their FXIII-A expression, just as the ongoing tissue damage and reorganization might be an inducer for a subset of alternatively activated macrophages.

It is important to emphasize that FXIII-A is also not correlated with the presence of a pathogen but with the microinflammatory environment. This finding is best reflected in chronic suppurative granulomatous mycoses. An increased number of hypertrophied FXIII-A-positive DCs with prominent dendrites were seen in paracoccidioidomycosis [47] and in chromoblastomycosis, of which some were colocalized with the pathogen itself [48].

In systemic histiocytic disorders, such as juvenile xanthogranuloma, xanthoma disseminatum, Erdheim-Chester disease, and dendrocytomas, FXIII-A, along with other markers (e.g., S100, CD1a, CD68, fascin, CD207, and CD35), is also used as a key diagnostic tool [49–51]. As reported previously, FXIII was coexpressed with CD68 in these diseases, allowing the conclusion that these cells are mostly phagocytes. However, the mechanisms behind its induction and the question as to whether FXIII-A has any role in these diseases remain unanswered.

### 5.2. FXIII-A in Neoplasms

The expression of FXIII has also been studied in solid tumors, but the specificity of the antibodies used for FXIII-A and the lack of multiple colabeling to characterize the FXIII-A-expressing cells might also lead to misinterpretation of these results with time.

Kaposi's sarcoma is a lymphangioproliferative disease caused by an HHV8 infection; it has 4 clinical presentations: classic, African endemic, iatrogenic immunosuppression related, and acquired immunodeficiency syndrome (AIDS) related. It was one of the first malignancies where FXIII-A became introduced as a diagnostic tool. The spindle-shaped FXIII-A-positive DCs around the small blood vessels suggested that they might play a role in the angioproliferative process [52]. Confirming the importance of these findings, FXIII-A is still a widely used marker in the diagnosis of Kaposi's sarcoma, but without any association to AIDS.

Another disease in which FXIII-A has stood the test of time and is used in the diagnosis is dermatofibroma (DF) where the spindle-shaped cells are FXIII-A positive but negative for CD34, in contrast to dermatofibrosarcoma protuberans (DFSP) where the cells are negative for FXIII-A and CD34 positive [53, 54]. The CD34 and FXIII-A-positive (CDa1−) marker combination has also been described in medallion-like dermal dendrocyte hamartomas, which is a benign congenital dermal lesion [55].

FXIII-A was also suggested to be a useful tool in differentiating between schwannomas neurofibromas and neurotized melanocytic nevi, which are common benign cutaneous neoplasms. In contrast to S100 protein, which was found to be expressed by tumor cells in all three diseases, FXIII-A was only found in neurofibromas (30–70% of cells within the tumors were positive for FXIII-A) [56]. However, whether the same cells were expressing S100 and FXIII-A proteins or FXIII-A positive cells were distinct ones infiltrating the tumor were not confirmed.

The expression of FXIII was examined in patients with tuberous sclerosis as well, where it was present in the stromal cells of the skin lesion [57]. It was suggested that cFXIII-A also takes part in fibrous tumor formation as an inducer of growth, such as a growth factor, based on its expression in fibrokeratomas, angiofibromas (adenoma sebaceum of Pringle), and oral fibrous hyperplasia [5].

A study of patients with oral squamous cell carcinoma suggested that the Leu allele of the FXIIIVal34Leu polymorphism, which decreases FXIII-A activity, is associated with an increased risk of squamous cell carcinoma, although without the increase in disease progression. Possibly both of these findings are due to a less porous fibrin network of thinner fibers that may facilitate tumor stroma formation and tumor cell proliferation but harbor tumor cells less effectively during metastasis formation [58]. In addition to this finding, in basal cell carcinoma and superficial malignant melanoma, an increase in the density of FXIII-A-positive DCs was associated with a low proliferative rate [59].

According to studies by our laboratory with the newest marker combinations and antibodies, in the various examined skin malignancies, FXIII-A-positive cells were almost exclusively tumor-associated macrophages. Tumor-associated macrophages are also polarized via the alternative pathway fitting into the concept that FXIII-A-positive macrophages are associated with tissue remodeling processes, thus being the prime pathological features in most of the malignancies. Importantly, FXIII-A-positive cells were found in high numbers in close association with fibrin deposits around tumor cells suggesting that ecFXIII-A with a possible macrophage source might be involved in tumor matrix formation [9, 60]. In line with this finding, an interesting study demonstrated that FXIII contributes to hematogenous metastasis formation, as the genetic elimination of FXIII-A significantly decreased lung metastasis formation in mice. As a possible explanation, FXIII was shown to enhance the early survival of embolic tumor cells by impeding natural killer cell function and protecting the newly localized tumor cells from natural killer cell-mediated lysis where the crosslinked fibrin matrices might be crucial [61].

Taking these data into consideration, to answer in full detail whether FXIII-A-positive macrophages support or inhibit tumor growth is an interesting topic for future studies.

### 5.3. FXIII-A in T-Cell Dermatoses

FXIII-A-positive cells showed increased numbers in skin samples from atopic eczema, psoriasis, lichen planus, spongiotic dermatitis, chronic graft versus host disease (GVHD), pityriasis alba, and mycosis fungoides. Introducing these diseases in more detail is beyond the scope of this review. In summary, these inflammatory diseases all have a notable accumulation of T cells, which led to the hypothesis that FXIII-A-positive cells might be able to cross talk with T cells through their dendrites and could present certain antigens. A possible FXIII-A-positive cell, lymphocyte interaction was observed in atopic dermatitis at the site of spongiosis and vesicle formation in the epidermis, while, in psoriatic plaques, an expanding population of FXIII-A-positive DCs was accompanied by endothelial and T lymphocyte expansion [64].

Deguchi et al. compared the number of antigen-presenting cells in atopic dermatitis, psoriasis, lichen planus, and GVHD. They found that there was only minimal change in the number of CD68+ macrophages in all samples compared to healthy skin. However, there was a significant increase in the amount of CD1a+, FXIII-A-positive cells in lichen planus, spongiotic dermatitis, and chronic GVHD, both in the dermis and in epidermis. In psoriasis, even though the number of FXIII-A-positive cells increased, it did not reach statistical significance, and, in acute GVHD, there was a significant decrease in these cells [72]. In a murine model, Yoo et al. demonstrated that, during acute GVHD, FXIII-A-positive DCs migrated to the superficial dermis and became hypertrophic and highly branched [75]. Additionally, in drug-induced toxic epidermal necrolysis (TEN), decreased numbers of FXIII-A-positive DCs were detected [65].

An increased number of FXIII-A-positive DCs were also described in pityriasis alba, suggesting that it could mark a subtle inflammation behind the postinflammatory hypopigmentation, but other causes besides FXIII accumulation were not excluded [76].

### 5.4. FXIII-A in Sclerotic Disorders

Scleroderma, and its localized form, morphea, is characterized by fibrosis involving the dermis and the subcutaneous tissue, with prominent ECM reorganization. Characterizing the cells involved in the lesions, it was found that FXIII-A positivity was lost in the papillary dermis but became prominent in the reticular dermis at the site of fibrosis. Interestingly, preceding the appearance of intense fibrosis, a reduction was also found in FXIII-A-positive cell numbers. This finding pointed to the presence of FXIII-A-positive cells that varied according to different stages of disease pathogenesis [77] and the fact that these cells might be important in fibrosis by crosslinking the newly formed ECM also found in scleroderma [66].

Interestingly, in a case of self-healing papular mucinosis, which is a milder form of sclerodermoid lichen myxedematosus, CD34+ or FXIII-A-positive DCs have been described next to mast cells, suggesting that these cells together could contribute to the dermal mucin deposition of the lesions [67].

## 6. FXIII-A in Wound Healing and Angiogenesis

Wounding is the most commonly observed pathological condition, marked by the presentation of damage-associated molecular patterns (e.g., ATP, HSP, HMGB1, hyaluronan fragments, DNA, and heparin sulfate). It can be the result of trauma/injury, impaired circulation, or necrosis with an infectious background. In the complex process of wound healing, cells and enzymes work together in a finely tuned system. Wound healing is divided into three stages: the inflammatory phase, proliferative phase, and maturation phase. During the inflammatory phase (1–3 days of wound healing), the clot forms, the inflammatory cascade is initiated, and neutrophils and macrophages are drawn to the wound site. Around day 4, fibroblasts, vessel formation, and ECM production overtake the scenery, and the transition to the proliferative phase occurs. From week 3, myofibroblasts, collagen remodeling, and wound contraction become characteristic of the maturation phase [78, 79].

The strongest piece of evidence supporting the role of FXIII-A in wound healing is that 14–36% of patients with FXIII-A deficiency experience impaired wound healing and abnormal scar formation [8]. Such impaired wound healing was also confirmed by using FXIII-A transgenic mice (deletion of the exon encoding the active site cysteine of the mouse FXIII-A). Inbal et al. found significant differences in maturity and organization of the wound between the controls, the FXIII-A-deficient and the FXIII-A-deficient recombinant FXIII- (rFXIII-) treated mice. Whereas the controls and the group treated with rFXIII had a normal mature wound healing process, the FXIII-A-deficient mice without treatment showed delayed reepithelialization, irregular fibrotic scars with ill-defined edges, discoloration, and necrotized fissures on histological slides [80]. A study of myocardial infarction (MI) in mice showed that FXIII correlates with predicted healing time and ventricular wall thickness. Nonetheless, in a different study, the association of decreased FXIII levels with wall rupture in patients after MI and its beneficial effect in decreasing vascular permeability during myocardial ischemia were noted [81–84].

These findings all support the complex roles of FXIII-A and strongly suggested that the observed differences were not simply related to the blood coagulation defect accounting for the acute, excessive hemorrhage of these patients. The most significant observation was that the impaired wound healing could be almost completely rescued with rFXIII-A. Therefore, we focus on pFXIII-A and ecFXIII-A to address the pathways in which FXIII-A could play a role. Nevertheless, it is important to emphasize that FXIII-A expressing alternatively activated macrophages [34, 37] are important in the clearance of wound debridement, with an increased number of scavenger receptors and the binding of apoptotic cells [3, 78, 85].

After tissue injury, pFXIII is rapidly activated by thrombin and calcium. Besides binding fibrin polymers together to form a mature clot, FXIII-A_2_^*∗*^ interacts with complement C3, integrating it in the fibrin clot and with this delaying fibrinolysis [23]. Importantly, FXIII-A_2_^*∗*^ can crosslink matrix components as well such as fibronectin, vitronectin, osteopontin, thrombospondin, collagen VI, and von Willebrand factor, which could further impact cell attachment [8, 86, 87] and migration [88]. In addition, FXIII-A_2_^*∗*^ could mediate endothelial cell-platelet interaction through *α*_v_*β*_3_-integrin (vitronectin receptor), regardless of its transglutaminase activity [89], thus making FXIII-A important in new vessel formation and remodeling. Inbal and Dardik described that the binding of FXIII-A_2_^*∗*^ to endothelial integrin *α*_v_*β*_3_ leads to *α*_v_*β*_3_-VEGFR-2 cross-linkage and a consequent VEGFR-2 activation. VEGFR-2 activation increased cell proliferation and survival via multiple pathways by upregulating Erg-1 and cJun transcription factors and downregulating thrombospondin-1 (TSP-1) on mRNA and protein levels [90]. TSP-1 is an antiangiogenic protein that by binding CD36 on the surface of epithelial cells induces their apoptosis. In transgenic TSP-1-overexpressing mice, delayed granulation tissue formation and delayed wound healing were observed [91]. Importantly, Dardik et al. also found that an antibody for *α*_v_*β*_3_-integrin (vitronectin receptor) blocked the binding of FXIII-A_2_^*∗*^ to fibroblasts, showing this receptor to be the target of the factor also on fibroblasts suggesting its role in the antiapoptotic effect of FXIII [92].

As demonstrated above, FXIII plays a central role in all stages of wound healing: whether it is clot formation, extracellular matrix formation, macrophage/monocyte activation, enhancement of fibroblast, and epithelial cell migration, FXIII is an essential part of the pathway. These findings make it a promising future treatment option in wound healing, which we will discuss later to a fuller extent.

## 7. Future Perspectives for FXIII-A in the Field of Dermatology

Based on the variety of processes in which FXIII-A is considered to have a role (Table 2), translating basic research into clinical practice is an interesting field with potential pathological and therapeutic relevance, which we aim to address in the following section.

### 7.1. Understanding FXIII-A-Positive Cells in Skin Lesions

Detection of FXIII-A in inflammatory and malignant diseases will unquestionably keep its place in dermatological diagnosis. However, more data and relevant experiments are needed to answer the long-lasting question as to whether FXIII-A is just a marker or also contributes to disease pathogenesis. This question is of particular importance in malignancies, where answering the level of contribution to the function of tumor-associated macrophages is as important as the role of ecFXIII-A in tumor matrix remodeling and could give FXIII-A a place in predicting tumor progression.

More and more data are anticipated regarding the origin, the functional properties, and the gene expression profiles of FXIII-A-positive cells with the improvement of the methods and techniques to work on ex vivo dermal immune cells. These techniques may allow us to study them, perhaps even at a single-cell level, without affecting their marker profiles and behaviors during their isolation and separation, such as antigen presentation.

### 7.2. Wound Treatment

Application of FXIII is the most promising in wound management, with a potential breakthrough already. Administration of rFXIII has been tested as a treatment option in various diseases, such as Crohn's disease, and in microvascular surgeries. During vascular grafting, the addition of rFXIII to the fibrin sealant resulted in a more desirable outcome than with the fibrin alone [93]. Moreover, topical application of rFXIII on heterotopic neonatal mouse heart allografts produced higher numbers of new vessels and increased contractility compared to the untreated mice, suggesting that rFXIII could potentially have a therapeutic effect in diseases where restoring circulation is essential [94]. It could have importance in dermatological diseases such as chronic venous and neuropathic ulcers, NL, and pyoderma gangrenosum, an autoinflammatory disease with severe ulceration. In line with these findings, the studies conducted so far on the application of FXIII as a treatment option in dermal wound treatment are promising. Accelerated wound surface reduction, shorter healing time, improved availability of granulation tissue, and decreased secretion, and bleeding tendencies were all reported following daily local administration of FXIII concentrate on clear, noninfected wounds of chronic venous leg ulcers and on pyoderma gangrenosum lesions [24, 95–99].

However, it should be noted that success is very dependent on the selection of the involved subjects. This selection is in regard to the background of the wound, other comorbidities, and genetic associations. These criteria are best reflected in the case of arterial-venous mixed disease patients [95] where none of the beneficial effects could be observed, which was contrary to chronic venous leg ulcers (CVLU). In CVLU, the Val34Leu, Tyr204Phe, and Pro564Leu polymorphisms of FXIII-A did not shown any relation to the prevalence of the ulcer, but Leu34 and Leu564 alleles were associated with smaller ulcer size [100–103].

### 7.3. FXIII-A in Connective Tissue Disorders and Rejuvenation

The ability of FXIII-A to affect collagen synthesis and ECM remodeling is also of potential interest. In particular, FXIII-A was already shown to be beneficial in diseases such as scleroderma. After systemic application of rFXIII-A, a decrease in stiffness of the skin and improved musculoskeletal symptoms were observed. This finding could be related to its possible effect in downregulation of collagen synthesis [104]. We also need to mention here that elevated pFXIII levels were found to promote inflammation and degenerative tissue remodeling in rheumatoid arthritis [105, 106]. This finding should be kept in mind and considered in the safety measurements of studies yet to come.

Platelet-rich plasma (PRP) is widely used for its beneficial effects in wound healing and rejuvenation via multiple actions, as it contains several different types of molecules such as growth factors and cytokines [107]. Some of the results from treatment of chronic wounds with PRP [107–109] are similar to those we have discussed in relation to local application of rFXIII in CVLU [95]. Thus far, there have been no reports, at least to our knowledge, about the FXIII-A content of PRP or its contribution to the effects of PRP. Therefore, further investigations are needed to assess complex cellular functions, the substrate profile of FXIII-A, and its involvement in the various steps of wound healing and rejuvenation.

## Figures and Tables

**Table 1:** Markers used most often during immunohistochemistry to distinguish cells in the dermis and epidermis and their coexpression with FXIII in normal skin and dermatological conditions.

Marker	Coexpression with FXIII+/− (%)	In dermatology	Function	Expressing cells	References
CD11c (CR4)	−	Normal skin	(i) Type I transmembrane glycoprotein (ii) Integrin *α*X (ITGAX) forms with ITGB2 a leukocyte specific integrin receptor (CR4)	(i) Myeloid dendritic cells	[25, 37, 45, 62]
	−	NL			
	−	GA			
	−	AD			
	−	Psoriasis			
	−	Stromal cell cc.			

CD1c (BDCA-1)	−	Normal skin	(i) Type I membrane glycoprotein (ii) Mediating the presentation of nonpeptide antigens to T cells	(i) Myeloid dendritic cells	[25, 37, 63]
	−	NL			
	−	GA			
	+ (25%)	MF			
	+ (25%)	Psoriasis			

CD45	+	Normal skin	(i) Protein tyrosine phosphatase (iii) Required for differentiation, growth, mitosis	(i) Bone marrow derived cells	[37, 45, 64, 65]
	−	AD			
	−	Psoriasis			

HLADR	+++ (85%)	Normal skin	(i) MHC class II receptor (ii) Antigen presentation to T cells	(i) Antigen presenting cells	[37, 45, 49, 62]
	+	AD			
	+	Psoriasis			
	−	Histiocytosis			

CD34	− − − − −	JLS Self-healing papular mucinosis DF DFSP Oral lichen planus	(i) Single chain transmembrane glycoprotein (ii) Regulating cell-cell adhesion and inhibits hematopoietic cell differentiation	(i) Hematopoietic progenitor cells (ii) Endothelial progenitor cells (iii) Vascular endothelial cells	[54, 66–69]

CD11b (MAC-1*α*)	−	Normal skin	(i) Integrin *α*M chain (ii) Part of C3 complement receptor (iii) Receptor for C3bi, fibrinogen, FX	(i) Myeloid cells (ii) Monocytes (iii) NK cells (weak)	[45]

CD14	+ (22%)	Normal skin	(i) Lipopolysaccharide receptor	(i) Monocytes (ii) Macrophages (iii) Myeloid dendritic cells	[45, 62]

CD83	−	Normal skin	(i) Immunoglobulin receptor (ii) Antigen presentation, T cell activation	(i) Mature dendritic cells	[25, 49]
	−	NL			
	−	GA			
		Histiocytosis			

CD205 (DEC205)	−	Normal skin	(i) C type lecithin type I membrane protein, part of the macrophage mannose receptor family (ii) Participates in antigen endocytosis	(i) Mature dendritic cells (ii) Thymic epithelial cells (iii) Monocytes	[37]

CD209 (ICAM-3, DC-SIGN)	+++ (98.8%) +++ (96%) +++ (93.5%)	Normal skin NL GA	(i) C type lecithin receptor type II (ii) Adhesion receptor, connection between DC-T cells and DC-endothelial, antigen receptor	(i) Tissue dendritic cells (ii) Macrophages (iii) Immature dendritic cells	[25]

CD208 (LAMP-3, DC-LAM)	−	Normal skin	(i) Type I lysosome associated membrane glycoprotein (ii) Maturation marker (upregulated by GM-CSF, IL-4, TNF*α*)	(i) Mature dendritic cells	[25, 37]
	−	NL			
	−	GA			

CD123 (IL3R*α* chain)	−	Normal skin	(i) Forming high affinity IL3 receptor (ii) Cell growth and differentiation	(i) Plasmacytoid dendritic cells (ii) Basophil granulocytes	[37, 70, 71]
	−	Tuberculoid leprosy			
	−	Lepromatous leprosy			

CD68	+++ (85.5%) ++ (64.33%) ++ (62.3%) − − − − +++	Normal skin NL GA AD Psoriasis Lichen planus Chronic GVHD Dermal dendrocytomas	(i) Type I lysosomal/endosomal associated membrane glycoprotein (ii) Binding low-density lipoprotein	(i) Monocytes (ii) Tissue macrophages (iii) Dendritic cells	[25, 48, 49, 53, 65, 72, 73]

CD1a (Leu-6)	−	Normal skin	(i) Type I membrane glycoprotein (ii) Lipid and glycolipid antigen presentation	(i) Langerhans cells (ii) Dendritic cells (iii) Cortical thymocytes	[25, 45, 49, 63, 65, 70–72]
	−	NL			
	−	GA			
	−	AD			
	−	Psoriasis			
	−	Lichen planus			
	−	Chronic GVHD			
	−	Leprosy			
	−	LCH/DC histiocytosis			

CD207 (langerin)	−	Tuberculoid leprosy	(i) Type II C-type transmembrane lecithin receptor (ii) Capturing antigens and inducing Birbeck granule formation	(i) Langerhans cells (ii) Dendritic cells	[49, 70]
	−	LCH			

CD36	+++ (92%)	Normal skin	(i) Class B scavenger receptor (ii) Binding long chain fatty acids, oxidized LDL, collagen types I, IV and V, and thrombospondin, as well as for apoptotic cells	(i) Endothelial cells (ii) Erythrocytes (iii) Platelets (iv) Monocytes (v) Macrophages (vi) Macrophage-derived dendritic cells	[45]

CD54 (ICAM-1)	− +	Normal skin Lichen planus	(i) Type I transmembrane glycoprotein (ii) Adhesion molecule, ligand for integrin	(i) Monocytes (ii) Macrophages (iii) Lymphocytes (iv) Activated endothelial cells (v) Granulocytes (vi) Dendritic cells	[45, 69]

CD163 (M130)	+++ (97.8%)	Normal skin	(i) Single chain transmembrane protein, hemoglobin/haptoglobin complex scavenger receptor, (ii) Signal transduction for proinflammatory cytokine production	(i) Mature tissue macrophage (ii) Blood monocyte	[17, 25, 37]
	+++ (77.1%)	NL			
	+++ (85.5%)	GA			

CD206 (MMR)	+++ (94.8%) ++ (52.2%) ++ (74.7%)	Normal skin NL GA	(i) Type I membrane protein, macrophage mannose receptor (ii) Mediating antigen endocytosis/phagocytosis	(i) Macrophages (ii) Dendritic cells (iii) Endothelial cells	[25]

S100	− − − − + (?) −	LHC/DC histiocytosis Chromoblastmycosis DF DFSP Neurofibroma Schwannoma	(i) Ca^2+^ binding protein regulation of protein phosphorylation, transcription factors (ii) Ca^2+^ homeostasis, the dynamics of cytoskeleton constituents, enzyme activities, cell growth and differentiation, and the inflammatory response	(Neural crest cells) (i) Langerhans cells (ii) Dendritic cells (iii) Macrophages (iv) Keratinocytes (v) Adipocytes (vi) Myoepithelial (vii) Chondrocytes	[48, 49, 54, 56, 68, 74]

NL = necrobiosis lipoidica; GA = granuloma annulare; AD = atopic dermatitis; GVHD = graft versus host disease; DF = dermatofibroma; DFSP = dermatofibrosarcoma protuberans; LCH = Langerhans cells histiocytosis; DC = dendritic cell; JLS = juvenile localized scleroderma; − < 20% expression, 20% < + < 50%, 50% < ++ <75% expression, +++ > 75% expression; ?: possible coexpression.

**Table 2:** Previously described/suggested mechanisms of action of factor XIII related to skin homeostasis and diseases.

Affected cells by factor XIII					
ECM	Monocytes	Macrophages	Dendritic cells	Fibroblasts	Endothelial cells
Mechanism of action					

(i) Crosslinking ECM components and complement C3 (ii) Crosslinking bacterial surface components (iii) Fibrin matrix formation around tumor cells (iv) Inducing tumor cell exit to the vasculature	(i) Dimerization of AT1 and mobilization of cells (ii) Facilitating entry into the artery wall (iii) Inducing receptor mediated phagocytosis (iv) Antiapoptotic (v) Promoting proliferation migration	(i) Translocation to the nucleus and macrophage activation (ii) Gene expression regulation (iii) Released cFXIII-A by damaged macrophages	(i) Induction of antigen presentation (ii) Induction of cytokine production (iii) Induction of mucin deposition	(i) Enhancing adherence and migration (ii) Antiapoptotic (iii) Promoting proliferation migration (iv) Homeostasis of collagen production	(i) Endothelial-platelet interaction (ii) Antiapoptotic (iii) Promoting proliferation and migration

References					

[1, 8–10, 23]	[1, 8–10, 23]	[1, 8–10, 23, 25, 37, 43, 62]	[1, 8–10, 23, 67, 72, 75–81]	[1, 5, 8–10, 23]	[1, 8–10, 23]

ECM = extracellular matrix.

## Abbreviations

FXIII: Factor XIII
FXIII-A: Factor XIII subunit A
FXIII-B: Factor XIII subunit B
FXIII-A2: Factor XIII subunit A as a dimer
pFXIII: Factor XIII in plasma
cFXIII: Factor XIII in cells
ecFXIII: Factor XIII in the extracellular space
pFXIII-A: Factor XIII subunit A in plasma as part of the tetramer or in dimer form
cFXIII-A: Factor XIII subunit A in cells as part of the tetramer or in dimer form
ecFXIII-A: Factor XIII subunit A in the extracellular space as part of the tetramer or in dimer form
rFXIII: Recombinant factor XIII
FXIII-A_2_^*∗*^: Activated factor XIII subunit A.

## References

[1] Mitchell J. L., Mutch N. J. (2016). Novel aspects of platelet factor XIII function. *Thrombosis Research*.

[2] Poon M.-C., Russell J. A., Low S. (1989). Hemopoietic origin of factor XIII A subunits in platelets, monocytes, and plasma. Evidence from bone marrow transplantation studies. *Journal of Clinical Investigation*.

[3] Ádány R., Bárdos H. (2003). Factor XIII subunit A as an intracellular transglutaminase. *Cellular and Molecular Life Sciences*.

[4] Collin M., Mcgovern N., Haniffa M. (2013). Human dendritic cell subsets. *Immunology*.

[5] Nemeth A. J., Penneys N. S. (1989). Factor XIIIa is expressed by fibroblasts in fibrovascular tumors. *Journal of Cutaneous Pathology*.

[6] Shubin N. J., Glukhova V. A., Clauson M. (2017). Proteome analysis of mast cell releasates reveals a role for chymase in the regulation of coagulation factor XIIIA levels via proteolytic degradation. *Journal of Allergy and Clinical Immunology*.

[7] Clark L. N., Elwood H. R., Uhlenhake E. E., Smoller B. R., Shalin S. C., Gardner J. M. (2016). Nuclear factor XIIIa staining (clone AC-1A1 mouse monoclonal) is a highly sensitive marker of sebaceous differentiation in normal and neoplastic sebocytes. *Journal of Cutaneous Pathology*.

[8] Muszbek L., Bereczky Z., Bagoly Z., Komáromi I., Katona É. (2011). Factor XIII: A coagulation factor with multiple plasmatic and cellular functions. *Physiological Reviews*.

[9] Shi D. Y., Wang S. J. (2017). Advances of coagulation factor XIII. *Chinese Medical Journal*.

[10] Schroeder V., Kohler H. P. (2016). Factor XIII: structure and function. *Seminars in Thrombosis and Hemostasis*.

[11] Nagy J. A., Henriksson P., McDonagh J. (1986). Biosynthesis of factor XIII B subunit by human hepatoma cell lines. *Blood*.

[12] Inbal A., Muszbek L., Lubetsky A. (2004). Platelets but not monocytes contribute to the plasma levels of factor XIII subunit A in patients undergoing autologous peripheral blood stem cell transplantation. *Blood Coagulation and Fibrinolysis*.

[13] Griffin K., Simpson K., Beckers C. (2015). Use of a novel floxed mouse to characterise the cellular source of plasma coagulation FXIII-A. *The Lancet*.

[14] Cordell P. A., Kile B. T., Standeven K. F., Josefsson E. C., Pease R. J., Grant P. J. (2010). Association of coagulation factor XIII-A with Golgi proteins within monocyte-macrophages: Implications for subcellular trafficking and secretion. *Blood*.

[15] Töröcsik D., Szeles L., Paragh G. (2010). Factor XIII-A is involved in the regulation of gene expression in alternatively activated human macrophages. *Thrombosis and Haemostasis*.

[16] Esnault S., Kelly E. A., Sorkness R. L., Evans M. D., Busse W. W., Jarjour N. N. (2016). Airway factor XIII associates with type 2 inflammation and airway obstruction in asthmatic patients. *Journal of Allergy and Clinical Immunology*.

[17] Takabayashi T., Kato A., Peters A. T. (2013). Increased expression of factor XIII-A in patients with chronic rhinosinusitis with nasal polyps. *Journal of Allergy and Clinical Immunology*.

[18] Kappelmayer J., Bacsko G., Birinyi L., Zakany R., Kelemen E., Adany R. (1995). Consecutive appearance of coagulation factor XIII subunit A in macrophages, megakaryocytes, and liver cells during early human development. *Blood*.

[19] Myneni V. D., Hitomi K., Kaartinen M. T. (2014). Factor XIII-A transglutaminase acts as a switch between preadipocyte proliferation and differentiation. *Blood*.

[20] Nurminskaya M., Kaartinen M. T. (2006). Transglutaminases in mineralized tissues. *Frontiers in Bioscience*.

[21] Nurminskaya M. V., Linsenmayer T. F. (2002). Immunohistological analysis of transglutaminase factor XIIIA expression in mouse embryonic growth plate. *Journal of Orthopaedic Research*.

[22] Orosz Z. Z., Katona É., Facskó A., Módis L., Muszbek L., Berta A. (2011). Factor XIII subunits in human tears; their highly elevated levels following penetrating keratoplasty. *Clinica Chimica Acta*.

[23] Schroeder V., Kohler H. P. (2013). New developments in the area of factor XIII. *Journal of Thrombosis and Haemostasis*.

[24] Komáromi I., Bagoly Z., Muszbek L. (2011). Factor XIII: Novel structural and functional aspects. *Journal of Thrombosis and Haemostasis*.

[25] Töröcsik D., Bárdos H., Hatalyák Z. (2014). Detection of factor XIII-A is a valuable tool for distinguishing dendritic cells and tissue macrophages in granuloma annulare and necrobiosis lipoidica. *Journal of the European Academy of Dermatology and Venereology*.

[26] Muszbek L., Adány R., Szegedi G., Polgár J., Kávai M. (1985). Factor XIII of blood coagulation in human monocytes. *Thrombosis Research*.

[27] Adany R., Kiss A., Muszbek L. (1987). Factor XIII: A marker of mono- and megakaryocytopoiesis. *British Journal of Haematology*.

[28] Henriksson P., Becker S., Lynch G., MacDonagh J. (1985). Identification of intracellular factor XIII in human monocytes and macrophages. *Journal of Clinical Investigation*.

[29] Adany R., Belkin A., Vasilevskaya T., Muszbek L. (1985). Identification of blood coagulation factor XIII in human peritoneal macrophages. *European Journal of Cell Biology*.

[30] Kahn D. R., Cohen I. (1981). Factor XIIIa-catalyzed coupling of structural proteins. *BBA - Protein Structure*.

[31] Asijee G. M., Muszbek L., Kappelmayer J., Polgár J., Horváth A., Sturk A. (1988). Platelet vinculin: a substrate of activated Factor XIII. *Biochimica et Biophysica Acta (BBA)*.

[32] Polanowska-Grabowska R., Gear A. R. L. (2000). Heat-shock proteins and platelet function. *Platelets*.

[33] Makogonenko E., Goldstein A. L., Bishop P. D., Medved L. (2004). Factor XIIIa incorporates thymosin *β*4 preferentially into the fibrin(ogen) *α*C-domains. *Biochemistry*.

[34] Törocsik D., Bárdos H., Nagy L., Ádány R. (2005). Identification of factor XIII-A as a marker of alternative macrophage activation. *Cellular and Molecular Life Sciences*.

[35] Swirski F. K., Nahrendorf M., Etzrodt M. (2009). Identification of splenic reservoir monocytes and their deployment to inflammatory sites. *Science*.

[36] Quatresooz P., Paquet P., Hermanns-Lê T., Piérard G. E. (2008). Molecular mapping of factor XIIIa-enriched dendrocytes in the skin (review). *International Journal of Molecular Medicine*.

[37] Zaba L. C., Fuentes-Duculan J., Steinman R. M., Krueger J. G., Lowes M. A. (2007). Normal human dermis contains distinct populations of CD11c^+^BDCA-1^+^ dendritic cells and CD163^+^FXIIIA^+^ macrophages. *The Journal of Clinical Investigation*.

[38] Geissmann F., Manz M. G., Jung S., Sieweke M. H., Merad M., Ley K. (2010). Development of monocytes, macrophages, and dendritic cells. *Science*.

[39] Jayo A., Conde I., Lastres P., Jiménez-Yuste V., González-Manchón C. (2009). Possible role for cellular FXIII in monocyte-derived dendritic cell motility. *European Journal of Cell Biology*.

[40] Criado P. R., Jardim Criado R. F., Sotto M. N., Pagliari C., Takakura C. H., Vasconcellos C. (2013). Dermal dendrocytes FXIIIA+ phagocytizing extruded mast cell granules in drug-induced acute urticaria. *Journal of the European Academy of Dermatology and Venereology*.

[41] Criado P. R., Criado R. F., Takakura C. F. (2013). Ultrastructure of vascular permeability in urticaria. *The Israel Medicine Association Journal*.

[42] Zhang L. J. (2015). Innate immunity. Dermal adipocytes protect against invasive Staphylococcus aureus skin infection. *Science*.

[43] Kradin R. L., Lynch G. W., Kurnick J. T., Erikson M., Colvin R. B., McDonagh J. (1987). Factor XIII A is synthesized and expressed on the surface of U937 cells and alveolar macrophages. *Blood*.

[44] Nickoloff B. J., Griffiths C. E. M., Gallo R. C., Salahuddin S. Z., Nakamura S. (1989). Factor XIIIa - Expressing dermal dendrocytes in AIDS-associated cutaneous Kaposi's sarcomas. *Science*.

[45] Cerio R., Griffiths C. E. M., Cooper K. D., Nickoloff B. J., Headington J. T. (1989). Characterization of factor XIIIa positive dermal dendritic cells in normal and inflamed skin. *British Journal of Dermatology*.

[46] Probst-Cousin S., Poremba C., Rickert C. H., Böcker W., Gullotta F. (1997). Factor XIIIa expression in granulomatous lesions due to sarcoidosis or mycobacterial infection. *Pathology—Research and Practice*.

[47] Pagliari C., Sotto M. N. (2002). Correlation of factor XIIIa+ dermal dendrocytes with paracoccidioidomycosis skin lesions. *Medical Mycology*.

[48] Sotto M. N., De Brito T., Silva A. M. G., Vidal M., Martins Castro L. G. (2004). Antigen distribution and antigen-presenting cells in skin biopsies of human chromoblastomycosis. *Journal of Cutaneous Pathology*.

[49] Jaffe R. (1999). The histiocytoses. *Clinics in Laboratory Medicine*.

[50] Bains A., Parham D. M. (2011). Langerhans cell histiocytosis preceding the development of juvenile xanthogranuloma: A case and review of recent developments. *Pediatric and Developmental Pathology*.

[51] Kazi N., Bernert R., Moussa C., Magro C. (2014). A case of generalized eruptive histiocytosis in a 23-year-old man. *Dermatology Online Journal*.

[52] Nickoloff B. J., Griffiths C. E. M. (1989). The spindle-shaped cells in cutaneous Kaposi's sarcoma. Histologic simulators include factor XIIIa dermal dendrocytes. *American Journal of Pathology*.

[53] Altman D. A., Nickoloff B. J., Fivenson D. P. (1993). Differential expression of factor XIIIa and CD34 in cutaneous mesenchymal tumors. *Journal of Cutaneous Pathology*.

[54] West K. L., Cardona D. M., Su Z., Puri P. K. (2014). Immunohistochemical markers in fibrohistiocytic lesions: Factor XIIIa, CD34, S-100 and p75. *American Journal of Dermatopathology*.

[55] Cheon M., Jung K. E., Kim H. S. (2013). Medallion-like dermal dendrocyte hamartoma: Differential diagnosis with congenital atrophic dermatofibrosarcoma protuberans. *Annals of Dermatology*.

[56] Gray M. H., Smoller B. R., McNutt N. S., Hsu A. (1990). Immunohistochemical demonstration of factor XIIIa expression in neurofibromas: a practical means of differentiating these tumors from neurotized melanocytic nevi and schwannomas. *Archives of Dermatology*.

[57] Penneys N. S., Smith K. J., Nemeth A. J. (1991). Factor XIIIa in the hamartomas of tuberous sclerosis. *Journal of Dermatological Science*.

[58] Vairaktaris E., Vassiliou S., Yapijakis C. (2007). Increased risk for oral cancer is associated with coagulation factor XIII but not with factor XII. *Oncology Reports*.

[59] Piérard-Franchimont, Arrese J., Nikkels A., Piérard G., Al-Saleh W., Delvenne P. (1996). Factor XIIIa-positive dendrocytes and proliferative activity of cutaneous cancers. *Virchows Archiv*.

[60] Bárdos H., Juhász A., Répássy G., Adány R. (1998). Fibrin deposition in squamous cell carcinomas of the larynx and hypopharynx. *Thrombosis and Haemostasis*.

[61] Palumbo J. S., Barney K. A., Blevins E. A. (2008). Factor XIII transglutaminase supports hematogenous tumor cell metastasis through a mechanism dependent on natural killer cell function. *Journal of Thrombosis and Haemostasis*.

[62] Turnock K., Bulmer J. N., Gray C. (1990). Phenotypic characterization of macrophage subpopulations and localization of factor XIII in the stromal cells of carcinomas. *The Histochemical Journal*.

[63] Fivenson D. P., Nickoloff B. J. (1995). Distinctive dendritic cell subsets expressing factor XIIIa, CD1a, CD1b and CD1c in mycosis fungoides and psoriasis. *Journal of Cutaneous Pathology*.

[64] Morganroth G. S., Chan L. S., Weinstein G. D., Voorhees J. J., Cooper K. D. (1991). Proliferating cells in psoriatic dermis are comprised primarily of T cells, endothelial cells, and factor XIIIa+ perivascular dendritic cells. *Journal of Investigative Dermatology*.

[65] Paquet P., Quatresooz P., Piérard G. E. (2003). Factor-XIIIa-positive dendrocytes in drug-induced toxic epidermal necrolysis (Lyell's syndrome): Paradoxical activation in skin and rarefaction in lymph nodes. *Dermatology*.

[66] Sung J. J., Chen T. S., Gilliam A. C., McCalmont T. H., Gilliam A. E. (2011). Clinicohistopathological correlations in juvenile localized scleroderma: Studies on a subset of children with hypopigmented juvenile localized scleroderma due to loss of epidermal melanocytes. *Journal of the American Academy of Dermatology*.

[67] Yokoyama E., Muto M. (2006). Adult variant of self-healing papular mucinosis in a patient with rheumatoid arthritis: Predominant proliferation of dermal dendritic cells expressing CD34 or factor XIIIa in association with dermal deposition of mucin. *Journal of Dermatology*.

[68] Abenoza P., Lillemoe T. (1993). CD34 and factor XIIIa in the differential diagnosis of dermatofibroma and dermatofibrosarcoma protuberans. *American Journal of Dermatopathology*.

[69] Cardozo A., Moura-Castro C., Figueiredo M., Cuzzi T., Ramos-e-Silva M. (2009). Oral lichen planus and dermal dendrocytes. *Actas Dermo-Sifiliográficas*.

[70] Hirai K. E., Aarão T. L. D. S., Silva L. M. (2016). Langerhans cells (CD1a and CD207), dermal dendrocytes (FXIIIa) and plasmacytoid dendritic cells (CD123) in skin lesions of leprosy patients. *Microbial Pathogenesis*.

[71] De Alvarenga Lira M. L., Pagliari C., De Lima Silva A. A., De Andrade H. F., Duarte M. I. S. (2015). Dermal dendrocytes FXIIIa+ Are essential antigen-presenting cells in indeterminate leprosy. *American Journal of Dermatopathology*.

[72] Deguchi M., Aiba S., Ohtani H., Nagura H., Tagami H. (2002). Comparison of the distribution and numbers of antigen-presenting cells among T-lymphocyte-mediated dermatoses: CD1a+, factor XIIIa+, and CD68+ cells in eczematous dermatitis, psoriasis, lichen planus and graft-versus-host disease. *Archives of Dermatological Research*.

[73] Ünver N., Freyschmidt-Paul P., Hörster S. (2006). Alterations in the epidermal-dermal melanin axis and factor XIIIa melanophages in senile lentigo and ageing skin. *British Journal of Dermatology*.

[74] Cerio R., Spaull J., Jones E. W. (1989). Histiocytoma cutis: a tumour of dermal dendrocytes (dermal dendrocytoma). *British Journal of Dermatology*.

[75] Yoo Y. H., Park B. S., Whitaker-Menezes D., Korngold R., Murphy G. F. (1998). Dermal dendrocytes participate in the cellular pathology of experimental acute graft-versus-host disease. *Journal of Cutaneous Pathology*.

[76] Carneiro F. R. O., do Amaral G. B., Mendes M. D., Quaresma J. A. S. (2014). Tissue immunostaining for factor XIIIa in dermal dendrocytes of pityriasis alba skin lesions. *Anais Brasileiros de Dermatologia*.

[77] De-Sá-Earp A. P., Do Nascimento A. P., Carneiro S. C., Porto L. C., Monte-Alto-Costa A. (2013). Dermal dendritic cell population and blood vessels are diminished in the skin of systemic sclerosis patients: Relationship with fibrosis degree and disease duration. *American Journal of Dermatopathology*.

[78] Sorg H., Tilkorn D. J., Hager S., Hauser J., Mirastschijski U. (2017). Skin wound healing: an update on the current knowledge and concepts. *European Surgical Research*.

[79] Childs D. R., Murthy A. S. (2017). Overview of wound healing and management. *Surgical Clinics of North America*.

[80] Inbal A., Lubetsky A., Krapp T. (2005). Impaired wound healing in factor XIII deficient mice. *Thrombosis and Haemostasis*.

[81] Nahrendorf M., Aikawa E., Figueiredo J.-L. (2008). Transglutaminase activity in acute infarcts predicts healing outcome and left ventricular remodelling: implications for FXIII therapy and antithrombin use in myocardial infarction. *European Heart Journal*.

[82] Vanhoutte D., Heymans S. (2008). Factor XIII: the cement of the heart after myocardial infarction?. *European Heart Journal*.

[83] López-Sendón J., Gurfinkel E. P., de Sa E. L. (2010). Factors related to heart rupture in acute coronary syndromes in the global registry of acute coronary events. *European Heart Journal*.

[84] Noll T., Wozniak G., McCarson K. (1999). Effect of factor XIII on endothelial barrier function. *Journal of Experimental Medicine*.

[85] Fairweather D., Cihakova D. (2009). Alternatively activated macrophages in infection and autoimmunity. *Journal of Autoimmunity*.

[86] Barry E. L. R., Mosher D. F. (1990). Binding and degradation of blood coagulation Factor XIII by cultured fibroblasts. *Journal of Biological Chemistry*.

[87] Laurens N., Koolwijk P., de Maat M. P. (2006). Fibrin structure and wound healing. *Journal of Thrombosis and Haemostasis*.

[88] Barry E. L. R., Mosher D. F. (1988). Factor XIII cross-linking of fibronectin at cellular matrix assembly sites. *Journal of Biological Chemistry*.

[89] Dardik R., Shenkman B., Tamarin I. (2002). Factor XIII mediates adhesion of platelets to endothelial cells through *α*v*β*3 and glycoprotein IIb/IIIa integrins. *Thrombosis Research*.

[90] Inbal A., Dardik R. (2006). Role of coagulation factor XIII (FXIII) in angiogenesis and tissue repair. *Pathophysiology of Haemostasis and Thrombosis*.

[91] Dardik R., Loscalzo J., Inbal A. (2006). Factor XIII (FXIII) and angiogenesis. *Journal of Thrombosis and Haemostasis*.

[92] Dardik R., Krapp T., Rosenthal E., Loscalzo J., Inbal A. (2007). Effect of FXIII on monocyte and fibroblast function. *Cellular Physiology and Biochemistry*.

[93] Dickneite G., Metzner H., Nicolay U. (2000). Prevention of suture hole bleeding using fibrin sealant: Benefits of factor XIII. *Journal of Surgical Research*.

[94] Dardik R., Leor J., Skutelsky E. (2006). Evaluation of the pro-angiogenic effect of factor XIII in heterotopic mouse heart allografts and FXIII-deficient mice. *Thrombosis and Haemostasis*.

[95] Wozniak G., Dapper F., Alemany J. (1996). Factor XIII in ulcerative leg disease: Background and preliminary clinical results. *Seminars in Thrombosis and Hemostasis*.

[96] Herouy Y., Hellstem M. O., Vanscheidt W., Schopf E., Norgauer J. (2000). Factor XIII-mediated inhibition of fibrinolysis and venous leg ulcers. *Lancet*.

[97] Hildenbrand T., Idzko M., Panther E., Norgauer J., Herouy Y. (2002). Treatment of nonhealing leg ulcers with fibrin-stabilizing factor XIII: a case report. *Dermatologic Surgery*.

[98] Peschen M., Thimm C., Wey A. (1998). Possible role of coagulation factor XIII in the pathogenesis of venous leg ulcers. *Vasa - Journal of Vascular Diseases*.

[99] Hoetzenecker W., Guenova E., Mitev V., Schanz S., Ulmer A., Fierlbeck G. (2013). Treatment of pyoderma gangrenosum with topical factor XIII. *European Journal of Dermatology*.

[100] Gemmati D., Tognazzo S., Serino M. L. (2004). Factor XIII V34L polymorphism modulates the risk of chronic venous leg ulcer progression and extension. *Wound Repair and Regeneration*.

[101] Gemmati D., Tognazzo S., Catozzi L. (2006). Influence of gene polymorphisms in ulcer healing process after superficial venous surgery. *Journal of Vascular Surgery*.

[102] Gemmati D., Federici F., Catozzi L. (2009). DNA-array of gene variants in venous leg ulcers: detection of prognostic indicators. *Journal of Vascular Surgery*.

[103] Tognazzo S., Gemmati D., Palazzo A. (2006). Prognostic role of factor XIII gene variants in nonhealing venous leg ulcers. *Journal of Vascular Surgery*.

[104] Jullien D., Souillet A. L., Faure M., Claudy A. (1998). Coagulation factor XIII in scleroderma. *European Journal of Dermatology*.

[105] So A. K., Varisco P.-A., Kemkes-Matthes B. (2003). Arthritis is linked to local and systemic activation of coagulation and fibrinolysis pathways. *Journal of Thrombosis and Haemostasis*.

[106] Raghu H., Cruz C., Rewerts C. L. (2015). Transglutaminase factor XIII promotes arthritis through mechanisms linked to inflammation and bone erosion. *Blood*.

[107] Fernandez-Moure J. S., Van Eps J. L., Cabrera F. J. (2017). Platelet-rich plasma: a biomimetic approach to enhancement of surgical wound healing. *Journal of Surgical Research*.

[108] Mohammadi M. H., Molavi B., Mohammadi S. (2016). Evaluation of wound healing in diabetic foot ulcer using platelet-rich plasma gel: a single-arm clinical trial. *Transfusion and Apheresis Science*.

[109] Jee C., Eom N., Jang H. (2016). Effect of autologous platelet-rich plasma application on cutaneous wound healing in dogs. *Journal of Veterinary Science*.

